# Structure and function of lipid droplet assembly complexes

**DOI:** 10.1016/j.sbi.2023.102606

**Published:** 2023-05-05

**Authors:** Tobias C. Walther, Siyoung Kim, Henning Arlt, Gregory A. Voth, Robert V. Farese

**Affiliations:** 1 Cell Biology Program, Sloan Kettering Institute, Memorial Sloan Kettering Cancer Center, New York, NY, 10065, USA; 2 Howard Hughes Medical Institute, New York, NY 10065, USA; 3 Pritzker School of Molecular Engineering, University of Chicago, Chicago, IL, 60637, USA; 4 Department of Chemistry, Chicago Center for Theoretical Chemistry, James Franck Institute, and Institute for Biophysical Dynamics, University of Chicago, Chicago, IL, 60637, USA

**Keywords:** Triacylglycerol, Sterol esters, Seipin, Endoplasmic reticulum, Lipid droplet, Phospholipids, Surface tension, Phase separation

## Abstract

Cells store lipids as a reservoir of metabolic energy and membrane component precursors in organelles called lipid droplets (LDs). LD formation occurs in the endoplasmic reticulum (ER) at LD assembly complexes (LDAC), consisting of an oligomeric core of seipin and accessory proteins. LDACs determine the sites of LD formation and are required for this process to occur normally. Seipin oligomers form a cage-like structure in the membrane that may serve to facilitate the phase transition of neutral lipids in the membrane to form an oil droplet within the LDAC. Modeling suggests that, as the LD grows, seipin anchors it to the ER bilayer and conformational shifts of seipin transmembrane segments open the LDAC dome toward the cytoplasm, enabling the emerging LD to egress from the ER.

## Introduction

Living organisms obtain energy from the environment to perform work and generate heat. Because energy sources are not always available, mechanisms have evolved to store metabolic energy. The primary molecule for storing energy in eukaryotes and some prokaryotes is triacylglycerol (TG), with fatty acyl chains of highly reduced carbons that are esterified to a glycerol backbone. In cells, TGs are stored in an oil phase within phospholipid monolayer—bound organelles called lipid droplets (LDs). LDs range in size from a few hundred nanometers to several microns in diameter and can store many millions of TG molecules. LDs may also store other neutral lipids, such as sterol-, retinol-, or waxesters, providing reservoirs for membrane synthesis or signaling functions. In this review, we focus on the molecular processes of TG storage.

TG synthesis in cells is catalyzed by acyl-CoA:diacylglycerol acyltransferases 1 and 2 (DGAT1 and DGAT2) [1,2]. Recently available molecular structures of DGAT1 suggest the enzyme joins acyl-CoA and diacylglycerol in a catalytic pocket deep within the ER membrane and releases its TG product directly into the ER membrane via a lateral gate [3,4]. Less is known about the actions of DGAT2, but alphafold2 [5] predictions suggest that its catalytic center localizes to the cytoplasmic surface of the ER. The ER-localized DGAT2 can also localize around LDs [6–9], suggesting it promotes local TG synthesis at LDs.

In bilayer membranes, TGs are thought to be soluble to a concentration of ~3 mol% [10]. Above this concentration, oil lenses form spontaneously and could destabilize ER membranes and proteins. Evidence suggests that specific protein machinery evolved to spatially organize and catalyze the formation of oil droplets at dedicated sites in the ER to prevent the unregulated formation of LDs. Recent discoveries have identified this ER-based protein machinery as the LD assembly complex (LDAC).

The components of LDACs were identified from genetic, biochemical, and cell biological experiments. Subsequently, cryo-EM structures and computational simulations have provided insights into LDAC structure and function. This review focuses on this progress and presents a model of LD formation that derives from these findings. We also highlight areas where molecular understanding is lacking.

## Identification of LDAC components

**Figure 1:**
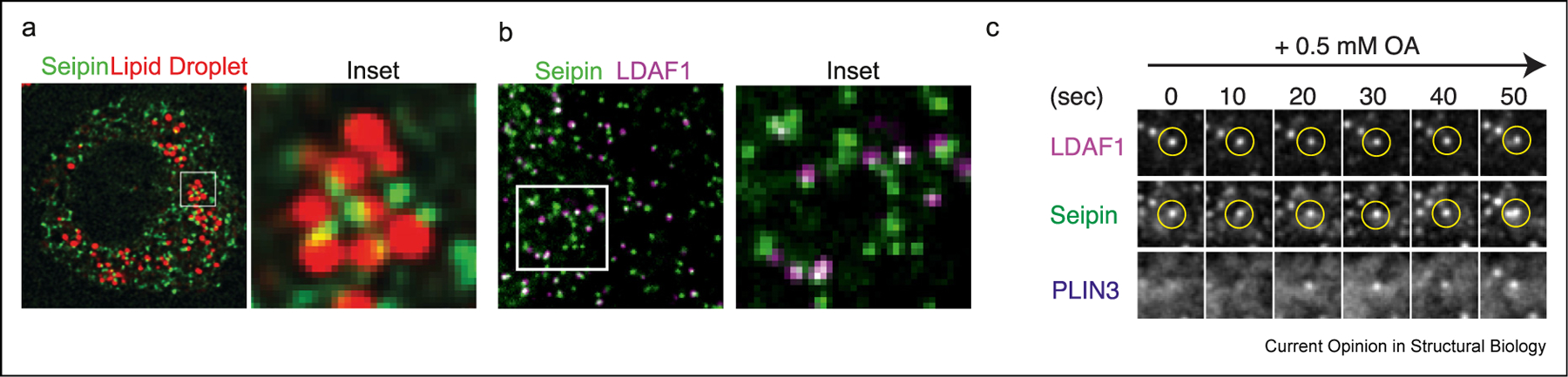
LDs form at seipin-containing LDACs in cells. **a.** Each cellular LD appears to co-localize with a single focus of seipin molecules in most cells studied, shown here for *Drosophila* S2 cells (see text for references). Reproduced with permission from the study by Wang et al. [21]. **b.** Seipin and LDAF1 colocalize in cells. Reproduced from Chung et al. [28]. **c.** LDs form at LDACs in cells. Detected by the sensitive PLIN3 protein marker, LDs form at ER locations where seipin oligomers and LDAF1 colocalize. Reproduced from the study by Chung et al. [28].

Mechanistic understanding of LD formation was enabled by the identification of LDAC protein components, with genetics providing the first insights. Human mutations in the *BSCL2* gene were identified to cause rare, congenital lipodystrophy, characterized by little or no adipose tissue [11]. Subsequently, systematic screens in yeast [12,13], fly and human cells [14,15], revealed that the seipin protein encoded by *BSCL2* is universally required for the formation of normally appearing LDs. Seipin is an evolutionarily conserved, small ~30–40 kDa ER protein with short N- and C-terminal segments in the cytosol, two transmembrane (TM) segments, and a lumenal domain. Numerous studies found that seipin forms an oligomeric complex in the ER membrane [16,17]. In cells with LDs, seipin complexes localize at the sites of LD formation [12,18–21] (Figure 1a), with each LD having a seipin focus at a membrane connection between the ER and the LD.

In the absence of seipin, LD formation is disorganized, with many relatively small LDs (~150-200-nm diameter) budding and detaching from the ER [21]. LDs that form with seipin deficiency have altered protein composition [18,21]. They also are deficient in phospholipid surfactants and, consequently, often coalesce to form larger, “supersized” LDs [22]. Seipin function is widely conserved in eukaryotic evolution. However, in some yeasts, including *S. cerevisiae,* seipin function is provided by two polypeptides, Fld1/Sei1 and Ldb16 [18,23,24].

The identification of seipin inspired the search for protein-interaction partners. While many potential interactors have been reported, only a few have been reproduced in multiple studies. In yeast, Sei1p interacts with Ldo45 and Ldo16, two proteins produced by alternative splicing from the same genome locus [25,26]. In human and fly cells, seipin interacts with LDAF1/TMEM159, which may be a distant orthologue of Ldo45p [27,28]. LDAF1 is a small protein with several hydrophobic segments, and most protein predictions suggest two hairpin segments that are inserted into the membrane with both N- and C-termini exposed to the cytoplasm [28]. Many seipin foci in human cells localize with LDAF1 (Figure 1b). In light microscopy images, only foci that contain both fluorescently tagged seipin and LDAF1 appear to form LDs, labeled by the sensitive LD surface marker PLIN 3 (Figure 1c). Because of this, the complex between functional seipin (e.g., seipin or Sei1p/Ldb16p) and LDAF1 or Ldo45p has been termed the LDAC [28]. LDAF1 seems to not be strictly required for LD formation but helps to optimize the process [28]. Highlighting their interaction, deletion of seipin in cells also results in the loss of the LDAF1 [28].

## Molecular structure of LDACs

**Figure 2:**
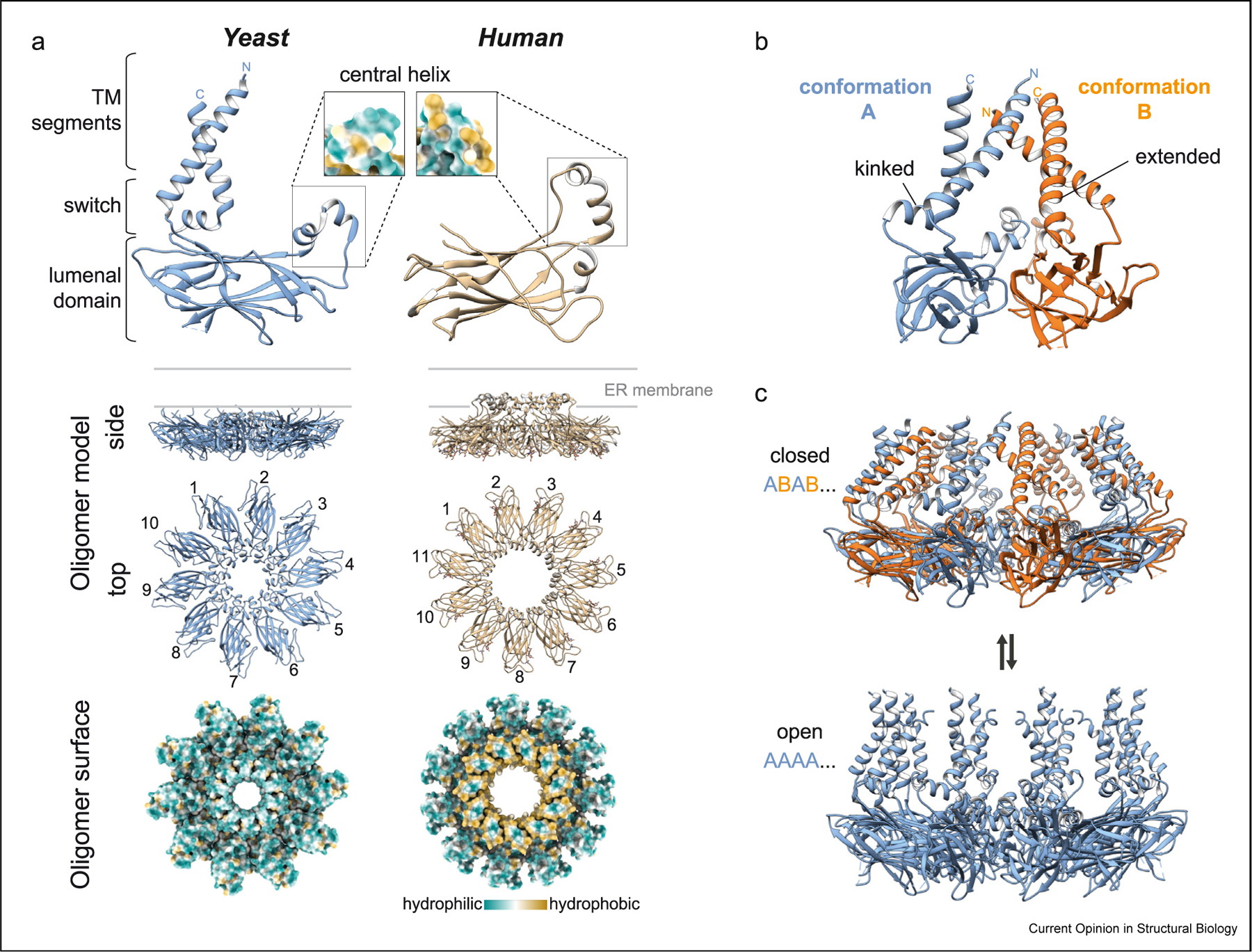
Overview of seipin structures of different species and predicted conformational changes in TM segments as determined by yeast seipin structure. **a.** Comparison of seipin cryo-EM structures from yeast and human. Detailed views of seipin monomers (top) or oligomeric complexes (middle and bottom) from yeast (PDB:7RSL) or human (PDB:6DS5). Central helices from yeast and human differ by hydrophibicity (see monomer inlays) and position relative to the ER membrane. The central helix of human seipin is hydrophobic and predicted to be embedded in the ER bilayer. **b.** Structure of yeast seipin TM segments shows two conformations in two adjacent monomers (PDB:7RSL), which are dictated by a conformational change in the switch region (kinked or extended) below the C-terminal TM segment. **c.** Comparison of yeast seipin cryo-EM structures shows two alternative TM conformations. Seipin TM segments adopt either a closed (ABAB ….) (PDB:7RSL) or open conformation (AAAA …) (PDB:7OXP). The open conformation is predicted to occur as LDs bud.

Seipin’s amino acid sequence is evolutionarily conserved, with two TM segments and an ER lumenal domain that, in humans, contains an N-linked glycosylation site. Mutations affecting the glycosylation site give rise to motor neuron disease [29]. The seipin protomers form an oligomeric complex that contains 10, 12, or 11 subunits in yeast, flies, and humans, respectively [23,30–32] (Figure 2a). Cryo-EM structures of the fly and human lumenal domains revealed a stable ring of lumenal domains, oriented parallel to the plane of the ER membrane [31,32]. Each domain has a β-sandwich fold that is reminiscent of lipid-binding proteins, such as NPC2. The human lumenal domain binds negatively charged lipids, such as phosphatidic acid, but the significance of this finding is unclear [32]. At the center of the lumenal ring, fly or human seipin lumenal domains project an α-helix that inserts into the ER membrane. In agreement with this, the amino acids on the surface of these helices are generally hydrophobic. In isolation, peptides from these helices bind to artificial LD surfaces [31]. Yeast seipin (Sei1p) has a similar lumenal domain structure [23,30], but the yeast oligomer lacks the equivalent hydrophobic helices in the center of the ring. Related functionality may be provided by Ldb16. This hypothesis is supported by crosslinking and mutational studies (23), but a structure with Sei1p in complex with Ldb16p has yet to be determined.

Evolutionary conservation of several polar amino acids in the central hydrophobic helix led to the hypothesis that this region interacts with a membrane protein. Indeed, two conserved serine residues in the central helix are required for the interaction of seipin with LDAF1 [28]. Seipin and LDAF1 were co-purified to apparent homogeneity in a stoichiometric complex that, unlike seipin alone, contains TG [28]. Structural analysis of this complex showed no EM densities for LDAF1, suggesting it might be flexible. Compared with the flat micelle for purified seipin alone, the micelle of seipin, LDAF1 and TG is curved towards the cytoplasm-facing side [28], possibly representing an early intermediate of LD formation.

The protein preparations used for the fly and human structures contained seipin’s TM segments, but densities for these regions of seipin were not observed in the cryo-EM maps. More recent structures for yeast Sei1 show the orientation of the Sei1 TM segments [23,30] (Figure 2a,b). In both reported structures, switch regions connect the lumenal domains to the TM domains that are tilted toward the center of the underlying ring structure. In one of the two structures [30], the C-terminal TM domains adopted two alternative conformations, A and B, that differ in the conformation of a switch region connecting the lumenal domain (Figure 2b). The A conformation is kinked, and the B conformation is more extended. Switching between different conformations of the TM segments results in a more closed (alternating A and B conformation) or open (all subunits in the A conformation) cage structure, suggesting the dome of the cage may be capable of opening toward the cytoplasm (Figure 2c).

The N- and C-terminal regions of seipin on either side of the ER membrane are evolutionarily less conserved. In yeast, the 10-amino acid cytosolic N-terminus is not required for seipin function [33]. For fly seipin, the N-terminal, cytoplasmic portion of the protein is longer and by itself sufficient to bind LDs [21]. Possibly, this segment interacts with LDs to aid in maintaining attachment of LDs to the ER. Both, N- and C-terminal regions of human seipin appear dispensable for function in LD formation [34].

## Insights into LDAC function from molecular dynamics simulations

Together, the lumenal ring and the TM segments of seipin form a large ~0.5-MDa, dome-shaped complex, with 20–24 TM segments (depending on species and not counting those of LDAF1 or yeast accessory proteins). How does this remarkable structure promote LD formation?

The LDAC structures currently available provide only snapshots of the seipin protein, and biochemical data testing LDAC function are limited. In the absence of such data, computational simulations, primarily coarse-grained molecular dynamics (MD) simulations, of seipin oligomers provide insights and illustrate likely functions of LDAC components.

**Figure 3:**
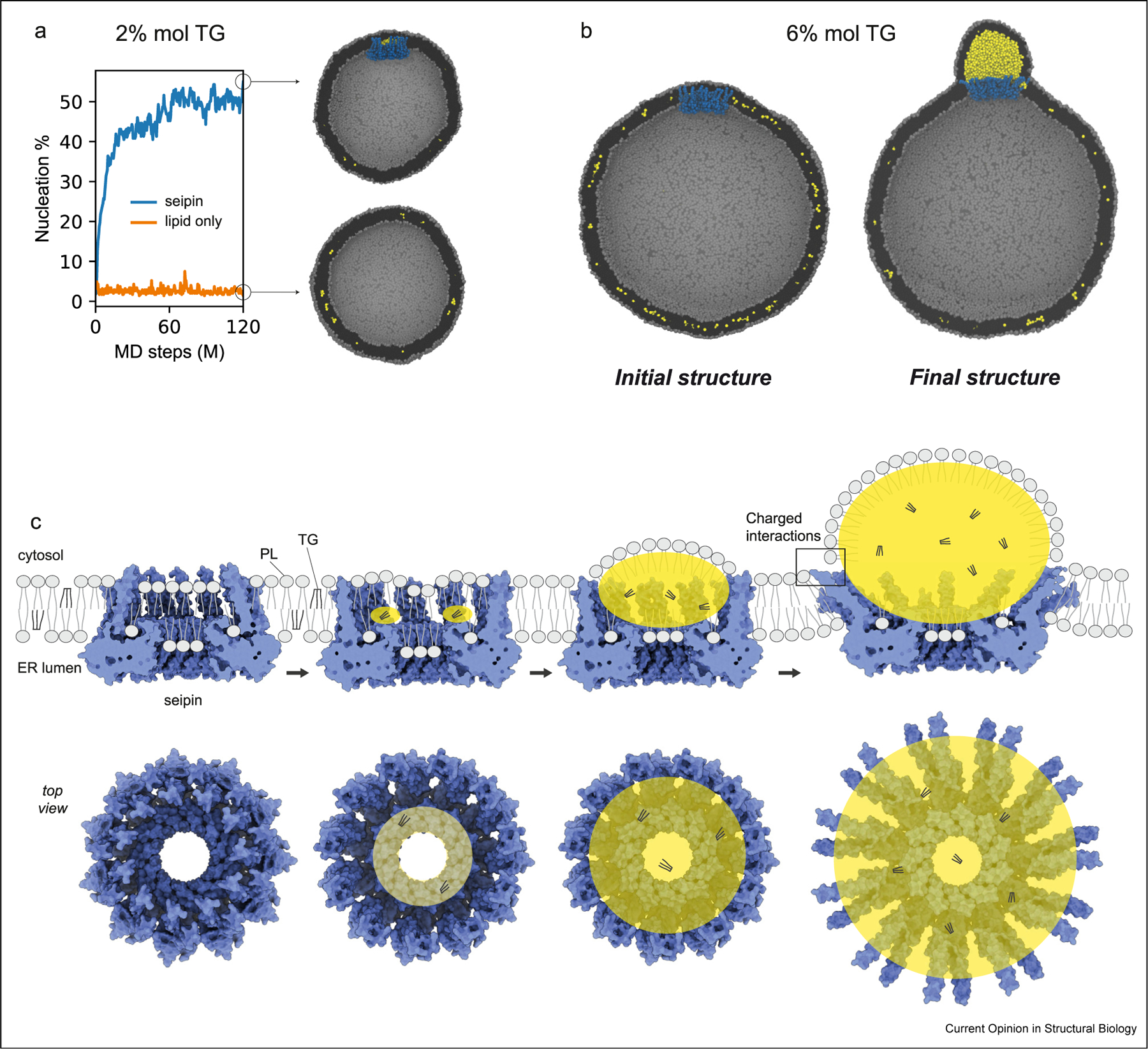
Molecular dynamics simulations showing how seipin affects TG phase separation. **a.** Seipin facilitates LD formation below the critical TG concentration. The spherical bilayer with a diameter of 40 nm contains 2% mol TG. **b.** MD simulations showing a large-scale LD formation in a spherical bilayer with a diameter of 60 nm and containing 6% mol TG. **c.** LD biogenesis model based on MD simulations data. Views from the top and side are shown. PL, phospholipid. a–b modified from results reported in the study by Kim et al. [34].

Seipin simulations of various resolutions have shown the LDAC and its central hydrophobic helices works as a nucleation site for TG, and forming an oil lens below the critical concentration (Figure 3a) [34–37]. In some simulations, the conserved serine residues in the central hydrophobic helices of human and fly seipin form hydrogen bonds with the glycerol groups of TGs, facilitating oil lens formation inside the LDAC complex. Similarly, the same conserved serine residues also interact with cholesterol esters in MD simulations, suggesting a broadly applicable mechanism for initial LD formation [36].

A large-scale MD simulation shows TG phase separation, LD growth, and ER-LD bridge formation at the LDAC (Figure 3b) [34]. Such MD simulations enhance our mechanistic understanding of LD biogenesis, as shown in Figure 3c. Conserved, positively charged residues at the end of N- and C-TM segments (exposed to the cytosol) are required for proper LD maturation. By interacting with the cytosolic leaflet, they maintain the structure and constrain the neck of the forming LD, allowing the budding of LDs from the ER with a high contact angle. Finally, simulations indicate that seipin TM segments open as an oil lens grows, agreeing with the findings from cryo-EM yeast structures.

## A hypothesis for LDAC-catalyzed formation of LDs from the ER

**Figure 4:**
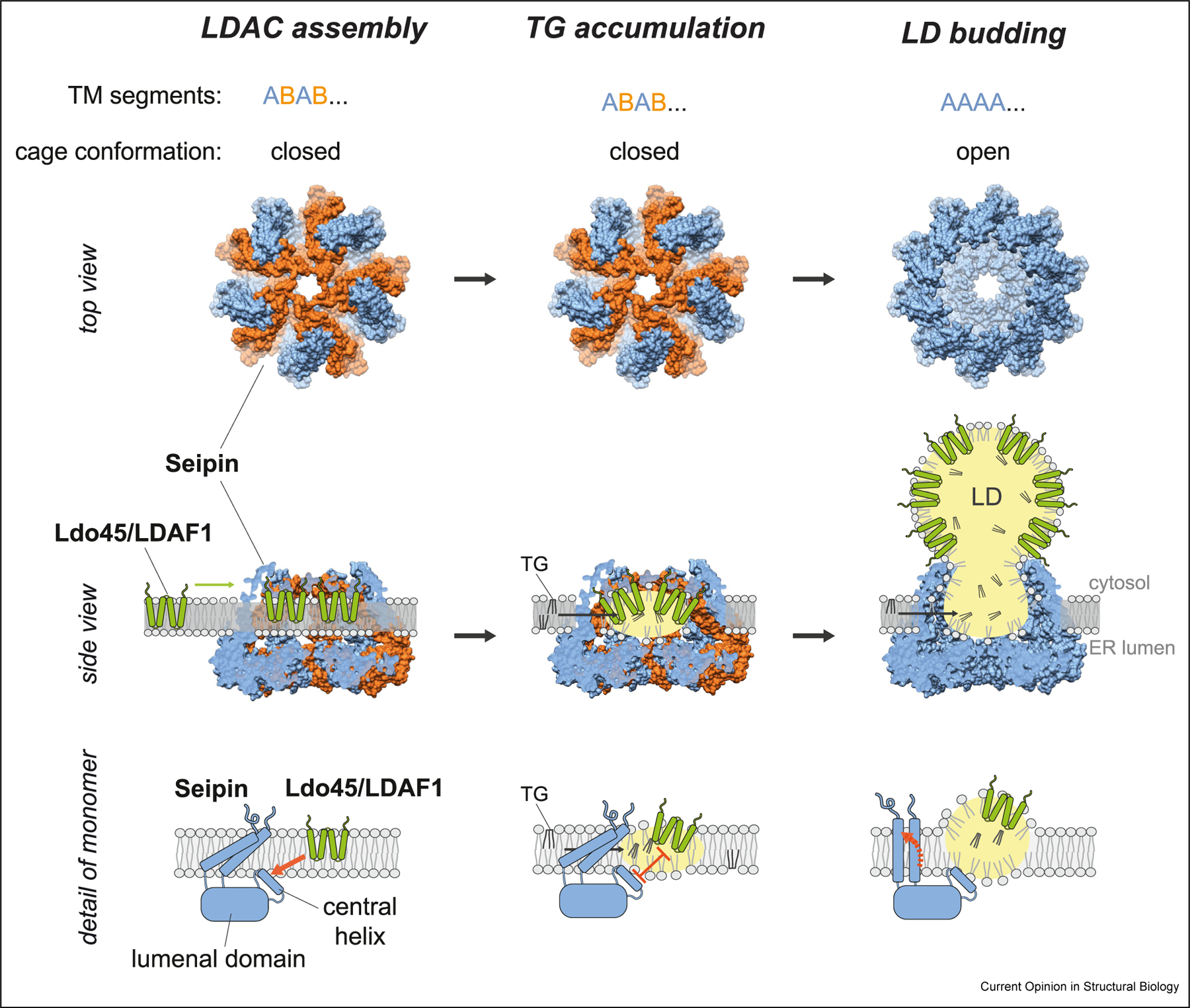
Hypothetical model for LD formation at LDACs in the ER. A model illustrating how the LDAC may promote LD formation within the ER membrane bilayer (figure adapted from the study by Arlt et al. [30]). See text for details. Views from the top (*en face*, from the cytosol) and side are shown. Bottom row shows detailed view of a seipin monomer embedded in the oligomeric structure. Note that the egressing LD shown is not drawn to scale due to space limitations; the forming LD in the schematic is shown as less than 15-nm diameter, whereas most newly formed LDs are 300-500-nm diameter.

Insights into the structures of LDAC, together with computational and experimental data, suggest a model for its function (Figure 4). LD formation is initiated by the synthesis of TG within the ER membrane [38]. TGs diffuse within the ER membrane bilayer, and phase separation does not happen when the TG concentration remains below ~3 mol%. In the closed conformation, the cage-like structure of seipin, with its multiple TM segments and hydrophobic helices, may provide a hydrophobic chamber for TG to phase-separate, possibly by allowing TG to preferentially (versus phospholipids) diffuse between TM segments into the chamber. Additional hydrophobic TM segments provided by LDAF1 (or Ldo proteins) may lie within the chamber. Inside the complex, the weakly polar TG molecules no longer interact extensively with phospholipids and thus can self-associate, leading to the nucleation of an oil phase, the rate-limiting step in the phase transition required for LD formation. In agreement with this idea, LDs form at lower TG concentrations in the ER when LDACs are present, than without them [28]. Once an initial LD forms, the dome-shaped LDACs may switch to an open conformation, opening up the dome to allow budding of the nascent LD toward the cytoplasm. The interaction of seipin and LDAF1 within the LDAC is released by the accumulating oil, and LDAF1 moves to the surface of the forming LD. The strong interactions of the seipin lumenal subunits provide a molecular anchor that maintains the connection of the forming LD with the ER. Charged residues on the cytoplasmic side of the seipin TM ensure that these sequences remain outside the hydrophobic part of the membrane as LDs form and may help to stabilize the neck connecting the ER and the LD [28,34]. In some species, this connection may also be supported by cytoplasmic N- and C-terminal extensions of seipin that interact with the base of the LD monolayer [21]. Consistent with the model, numerous studies show most LDs have a single adjacent seipin focus, and EM has revealed a 10-15-nm diameter neck at LD-ER connections [19].

As TG synthesis continues and TGs diffuse within the ER bilayer, more TGs partition into the forming LD by a process analogous to Ostwald ripening. Therefore, the nascent LD grows. During the process, more LDAF1 molecules, as well as selected other cargoes, target the growing surface of LDs. Other ER proteins are likely restricted [39], which may serve to control the initial LD surface protein composition. Still other proteins target LD surfaces at later time points, either from ER-LD membrane connections [7,39] or from the cytoplasm [40,41].

## Open issues

Structural biology and computational approaches have enabled great progress into understanding LD formation. Nevertheless, many issues remain unsettled.

First, a structural model of a complete LDAC, including accessory proteins, such as LDAF1 or Ldo45p, is lacking and is key for testing the current model for LD formation.

Also unclear is whether LDACs are dedicated to formation of TG-rich LDs or can catalyze formation of LDs containing other neutral lipids such as CE or retinol esters. Recent studies have explored this question and provided evidence that seipin is required for forming mostly CE-containing LDs [36,42].

Most importantly, the presented model has not been experimentally tested in many aspects, and other possibilities persist. For instance, seipin may be involved in lipid transfer [32] or Ca^2+^ homeostasis [43] or serve as a scaffold protein for lipid synthesis enzymes [44]. In yeast, additional factors, such as Pex30, appear to be localized to LDACs [45,46], but their roles are not yet well understood. Pex30 and related ER shaping proteins, such as reticulons or REEP proteins, may facilitate LD formation by altering local ER membrane curvature. Current evidence clearly indicates that LDs can form at ER tubules, but whether they form at ER sheets remains uncertain. However, at least in yeast, LDs can form at the nuclear envelope, demonstrating that, at least under some circumstances, LDs can form from a membrane sheet.

How LD formation relates to the numbers of LDACs in cells is also unclear. For example, cells may have many hundreds of LDACs and utilize only a small fraction for LD formation. What determines which or how many LDACs are utilized? Additionally, some prokaryotes, such as *Rhodococcus* and *Mycobacteria* [47], have LDs that bud from the plasma membrane, apparently without seipin or LDACs. How LD formation occurs in these species is uncertain.

Now that seipin and LDACs have been established as sites of cellular LD formation in the ER, studies can be directed to test LD formation models mechanistically. Additionally, determing LDAC structures at different stages of LD formation is now essential.

## Data Availability

No data were used for the research described in the article.
